# The performance of the EQ-5D-3L in screening for anxiety and depressive symptoms in hospital and community settings

**DOI:** 10.1186/s12955-021-01731-x

**Published:** 2021-03-19

**Authors:** Hilary Short, Fatima Al Sayah, Arto Ohinmaa, Jeffrey A. Johnson

**Affiliations:** 1 2-040 Li Ka Shing Centre for Health Research Innovation, School of Public Health, University of Alberta, Edmonton, AB T6G 2E1 Canada; 2 3-267 Edmonton Clinic Health Academy, School of Public Health, University of Alberta, Edmonton, AB Canada

**Keywords:** Screening, Depression, Anxiety, EQ-5D-3L, Hospital, Community setting

## Abstract

**Background:**

To examine the performance of the EQ-5D-3L in screening for anxiety and depressive symptoms in hospital and community settings compared to other patient-reported screening tools.

**Methods:**

Data from a prospective cohort of patients discharged from general internal medicine wards from two hospitals in Edmonton, Alberta were used in this study. Two waves of measurements (discharge and 90-days post-discharge) were analyzed. The performance of the EQ-5D-3L was compared to other self-report screening tools: Generalized Anxiety Disorder 2-item questionnaire was used to categorize anxiety symptoms into absent (< 3) and present (≥ 3), and the Patient Health Questionnaire 9-items was used to categorize depressive symptoms by two severity cut-points: no (< 10) vs. mild (≥ 10), and no (< 15) vs. moderate-severe (≥ 15). Performance of EQ-5D-3L in screening for anxiety and depressive symptoms was evaluated using receiver operating curve (ROC) analysis.

**Results:**

Average age of participants (n = 493) was 62.9 years (SD 18.6); 51% were female. At discharge, 30.0% screened positive for mild and 12.8% for moderate-severe depressive symptoms, while 27.6% screened positive for anxiety symptoms. For co-morbid symptoms, 17.1% screened positive for anxiety and any depressive symptoms, while 10.8% for anxiety and moderate-severe depressive symptoms. While the EQ-5D-3L had limited screening ability in hospital, the anxiety/depression dimension performed well in the community setting (90-days post-discharge) in screening for anxiety (area under ROC 0.79), depressive symptoms (any: 0.78, moderate-severe: 0.84), and a combination of both (any: 0.86; moderate-severe: 0.91).

**Conclusions:**

The EQ-5D-3L anxiety/depression dimension could be a useful tool in screening for anxiety and depressive symptoms in community settings compared to other self-report screening tools. The usefulness of the EQ-5D-3L as a screening tool in other settings and populations is warranted.

## Background

Physical and mental health have a complex, bidirectional relationship [1]. Poor mental health is associated with increased risk of physical illness and poor physical health is related to higher risk for mental disorders, most commonly depression and anxiety [1]. Mental disorders are common and rank as the leading cause of disability in Canada [2]. The chance of having a mental illness in a Canadian lifetime is 1 in 5 [2]. Estimated expenditure on non-dementia-related mental healthcare in Canada was $15.8 billion in 2015 [3], yet one-third of adults may not have their mental health care needs fully met [4]. Healthcare systems should be strengthened to improve the delivery on mental health care, particularly in ‘first point of contact’ settings such as hospitals and primary care.

Significant mental disorders occur in about 30% of hospitalized inpatients [5], however, less than 5% of admissions receive a mental health assessment [6–8]. Inpatient care often focuses on treating patients’ presenting illness or injury and discharging them as quickly as possible [9]. Referrals to psychiatric consultation or liaison services are low [10], and inadequate identification and management of mental disorders has significant implications [11]. Hospitalized patients with undiagnosed, and therefore untreated, mental disorders have poorer outcomes [9, 12, 13], increased mortality [14], extended hospitalization [9, 12, 13, 15], greater risk for hospital readmission [11–13], greater use of medical services [16, 17], and reduced compliance with treatment [9].

It has been estimated that 80% of people with major depressive disorder are treated entirely in the primary care setting, resulting in depression being one of the most prevalent and fastest rising disorders in primary care [18]. Additionally, generalized anxiety disorder is the most frequent anxiety disorder in primary care, with 6% to 22% of primary care patients complaining of anxiety problems, which suggests that these patients are high users of primary care resources [19–21]. However, mental disorders are frequently under-diagnosed in primary care [22]. Studies show that primary care physicians recognize depressive episodes less than 50% of the time [23] and identify anxiety and mood disorders at “chance levels of probability” [24, 25]. Many individuals may suffer from mental disorders for years before receiving appropriate treatment, resulting in significant morbidity for those who suffer from these disorders in addition to social and economic costs [18].

Hospitalization and primary care visits represent unrecognized opportunities to improve both mental and physical health outcomes [9]. Evidence shows that common mental disorders can be effectively treated in people with a physical health condition [1], and integrated mental healthcare positively impacts physical health outcomes [26] and reduces services use and healthcare costs [27, 28]. However, diagnosing mental disorders in the physically unwell can be challenging for the examining physician [29]. Furthermore, hospital and primary care physicians may view mental disorders as out of their scope of practice and a problem to be managed by other health care professionals [11].

Given the high rate of under-diagnosed mental disorders, a suitable screening instrument for the inpatient and outpatient environment to aid the diagnostic process would be useful. While screening measures do not replace a clinical diagnosis, an effective screening measure can prompt health care providers to further assess patients’ mental health and refer patients that may have been overlooked to a mental health professional. In Canadian healthcare settings, there is no consistent approach of screening for mental health symptoms. Patient-reported outcome measures (PROMs) of general health status have been increasingly used for routine outcome measurements in health systems in Canada and internationally [9]. The EQ-5D, which includes an item assessing anxiety and depression, is a commonly used generic measure in routine outcome measurement initiatives. It has been suggested that a single question on depressed mood can detect 85–90% of patients with major depressive disorder [30]. More elaborate screening tools have not attained broad acceptance by general physicians due to the time involved in administering and interpreting them, as well as their focus on the diagnosis and detailed diagnostic criteria of mental illness. The *Diagnostic and Statistical Manual of Mental Disorders*, Fourth Edition (*DSM-IV*) is of limited utility for primary care or physicians or internists [25, 31] who may not have the time or inclination to determine whether a patients’ emotional distress might meet criteria for a mental illness. In fact, it’s been shown that half of primary care physicians often do not use the DSM-IV diagnostic criteria when diagnosing depression [32].

Within Alberta, Canada, the healthcare system endorsed and embedded the EQ-5D in *Connect Care*, the province-wide hospital-based electronic patient medical information system, and is widely collected across the healthcare system. Additionally, the EQ-5D has been adopted for clinical use within Alberta’s Primary Care Networks. This tool offers an opportunity for a standardized approach to routine mental health screening if it is found fit for this purpose [33]. Given the crucial need for improved anxiety and depression detection, we sought to examine whether the EQ-5D might be suitable as a mental health screening tool, compared to other self-report screening tools, in the hospital setting as well as after discharge in the community setting.

## Methods

### Data source

This study was a secondary analysis of data from a prospective cohort of patients 18 years of age and older who were being discharged from seven general internal medicine wards from two hospitals in Edmonton (University of Alberta and Royal Alexandra hospitals) between October 2013 and November 2014 [34]. Patients were excluded if they did not live in the province, had severe cognitive impairment, were deemed by their attending physician to have fore-shortened life expectancy that would preclude 90-day follow up, or were transferred to or from a long-term care facility, another inpatient service or another acute care hospital [34]. Data collected at the time of discharge was used to evaluate the EQ-5D-3L screening performance in the hospital setting, and data from 90-days post discharge, collected by telephone interview, was used for the community setting.

### Measures

The EQ-5D-3L is a generic preference-based measure of health-related quality of life. It measures five dimensions of health (mobility, self-care, usual activities, pain/discomfort, and anxiety/depression), by three levels of perceived problems: no problems “1”, some/moderate problems “2”, extreme problems/unable to/confined to bed “3” [35]. The EQ-5D-3L describes 243 unique health states defined by combining one level from each of the five dimensions; 11111 is the best health state (no problems on any of the dimensions) and 33333 is the worst health state (extreme problems on all of the dimensions) [35]. The reference period of the EQ-5D is “today”. The EQ-5D-3L also includes a visual analogue scale (EQ-VAS), which represents the respondent’s self-rated health ‘today’ on a scale ranging from 0 “worst imaginable health state” to 100 “best imaginable health state” [35]. Population value sets are used to derive index scores for each health state [36]. We used the EQ-5D-3L Canadian value set to calculate index scores in this study [37].

The performance of the EQ-5D-3L (anxiety/depression dimension and index score) in screening for anxiety and depressive symptoms was evaluated in comparison to two self-report screening instruments: The Generalized Anxiety Disorder 2-item (GAD-2) questionnaire and the Patient Health Questionnaire 9-items (PHQ-9).

GAD-2 assesses the presence and frequency of anxiety symptoms “over the last 2 weeks” [38]. It includes two items that are scored from 0 (not at all) to 3 (nearly every day), with higher scores indicating higher severity of anxiety symptoms. A total score was generated by summing the two items (range 0–6) and presence of anxiety symptoms is indicated by a cut-off point of ≥ 3 [38]. A score of 3 points is the preferred cut-off for identifying possible cases, and in which further assessment for generalized anxiety disorder is warranted [39]. The total score was categorized as present (GAD-2 ≥ 3) vs. absent (GAD-2 < 3) anxiety symptoms. The sensitivity and specificity of the GAD-2 in identifying generalized anxiety disorder in primary care were reported to be 86% and 83%, respectively [38]. Despite the GAD questionnaire being a commonly used screening instruments for anxiety symptoms, there are no studies reporting its usefulness in hospital or inpatient settings.

The PHQ-9 measures the presence and frequency of depressive symptoms “over the last 2 weeks”. All 9 items are scored from 0 (not at all) to 3 (nearly every day), with higher scores indicating higher severity of depressive symptoms. A total score is generated by summing the scores of the 9 items (range 0–27) [40]. A meta-analysis on the use of the PHQ-9 in screening for major depressive disorder in primary care and obstetrics-gynecology patients indicated that the pooled sensitivity and specificity estimates of the PHQ-9 were 78% and 87%, respectively [39]. A validation study of the PHQ-9 in assessing major depressive disorder during inpatient spinal cord rehabilitation reported 100% sensitivity and 84% specificity [41]. The total PHQ-9 score was categorized into two severity levels:

- Any depressive symptoms (PHQ-9 ≥ 10) vs. absent depressive symptoms (PHQ-9 < 10) [40]
- Moderate-severe depressive symptoms (PHQ-9 ≥ 15) vs. absent moderate-severe depressive symptoms PHQ-9 < 15) [40]

### Statistical analysis

Descriptive statistics were computed for demographic variables in the sample at the time of discharge. Chi-square tests and analysis of variances (ANOVA) were used as appropriate to examine the differences between healthy and anxious and/or depressed groups in mean index scores and distribution of problems in the anxiety/depression dimension. The EQ-5D-3L index score was categorized into tertiles. The anxiety/depression dimension has three levels; 1 = not anxious or depressed, 2 = moderately anxious or depressed, 3 = extremely anxious or depressed and was used as such in the analysis. In addition to examining anxiety and depressive symptoms based on GAD-2 and PHQ-9 as separate variables, we created two composite variables, one for each cut-off point of PHQ-9 (any; moderate-severe), as follows:

- Absent anxiety and depressive symptoms
- Present anxiety and no depressive symptoms
- Absent anxiety and present depressive symptoms
- Present anxiety and depressive symptoms

Area under receiver operating curve (AUROC) analysis was used to examine the performance of the EQ-5D-3L components (anxiety/depression dimension and index score) in screening for anxiety and depressive symptoms. Sensitivity, specificity, positive (LR+) and negative (LR−) likelihood ratios for presence of anxiety symptoms and the two severity levels of depressive symptoms at each cut-point, as well as an overall AUROC with 95% confidence interval (CI) were calculated to identify the optimal threshold for each of the examined components. AUROC values were interpreted as follows: ≤ 0.5 non-informative, 0.5 < AUROC ≤ 0.7 less accurate, 0.7 < AUROC ≤ 0.9 moderately accurate, 0.9 < AUROC < 1.0 highly accurate, and AUROC = 1 perfect test [42]. All analyses were performed using STATA 14.2 [43].

## Results

### General characteristics of participants

Of 720 eligible patients discharged from the medical wards during the study period, 493 enrolled (69% response rate). At 90-days post-discharge, there was 19% loss to follow-up (N = 402). Average age of participants at the time of discharge from hospital (N = 493) was 62.9 years (SD 18.6), half were female (51%), 82% were White, 49% were retired, and over half (58%) were single, widowed or separated. Nearly half (46%) had more education than a high school diploma, and more than two thirds (68%) had an annual household income of less than $80,000CAD. On average participants had 4.9 comorbidities (SD 2.8) and reasons for hospitalization varied significantly (Table 1).

**Table 1:** Sample characteristics at baseline

Mean ± SD or N (%)	Baseline/discharge (N = 493)
Age (years)	62.9 ± 18.6
Sex (female)	250 (51%)
*Marital status*	
Married/common-law	209 (42.2%)
Single/divorced/widowed	286 (57.8%)
*Smoking status*	
Current	118 (23.8%)
Former	218 (44.0%)
Never	159 (32.1%)
Education level	
< high school	188 (38.0%)
High school diploma	78 (15.8%)
> high school	229 (46.3%)
Employment status (retired)	243 (49.1%)
*Total household income $CAD*	
< 80,000	337 (68.2%)
≥ 80,000	90 (18.2%)
*Living situation (prior to admission)*	
Home without any support	289 (53.4%)
Home, informal caregiver	90 (18.2%)
Home, with homecare	76 (15.4%)
Home, with live-in caregiver	3 (0.6%)
Assisted living/lodge	37 (7.5%)
*Ethnicity*	
White	407 (82.2%)
Other	81 (16.3%)
*Reason for hospitalization*	
Heart failure	50 (10.1%)
Pneumonia/empyema/lung abscess	49 (9.9%)
Diabetes	26 (5.3%)
Urinary tract infection	26 (5.3%)
Venous thromboembolic disease (PE/DVT)	26 (5.3%)
Cellulitis/decubitus ulcers	16 (3.2%)
Sepsis	12 (2.4%)
ETOH or street drug intoxication/withdrawal	12 (2.4%)
Gastroenteritis/colitis	12 (2.4%)
Cancer	11 (2.2%)
Acute kidney injury	11 (2.2%)
Pancreatitis	10 (2.0%)
Other	234 (52.7%)
Number of comorbidities	4.9 ± 2.8

At the time of discharge, the mean GAD-2 score was 1.7 (SD 1.8), and 28% screened positive for anxiety symptoms (GAD-2 ≥ 3), while the mean PHQ-9 score was 7.4 (SD 5.8), and 30% screened positive for any depressive symptoms (PHQ-9 ≥ 10), and 12.8% screened positive for moderate-severe depressive symptoms (PHQ-9 ≥ 15). Further, 17% screened positive for co-morbid anxiety and any depressive symptoms (GAD-2 ≥ 3 and PHQ-9 ≥ 10), and 11% screened positive for co-morbid anxiety and moderate-severe depressive symptoms (GAD-2 ≥ 3 and PHQ-9 ≥ 15). The mean EQ-5D-3L index score was 0.69 (SD 0.21), with 42% reporting some to extreme (levels 2–3) problems on the anxiety/ depression dimension (Table 2).

**Table 2:** Anxiety, depression, and health status

Mean ± SD or N (%)	Hospital Setting (N = 493)	Community Setting (N = 402)
*EQ-5D-3L*		
Anxiety/depression dimension (levels 2–3)	209 (42.2%)	108 (26.9%)
Index score	0.69 ± 0.21	0.77 ± 0.22
VAS	63.4 ± 18.9	71.4 ± 19.4
*PHQ-9*		
Total score	7.4 ± 5.8	4.0 ± 4.6
≥ 10	148 (30.0%)	49 (12.2%)
≥ 15	63 (12.8%)	14 (3.5%)
*GAD-2*		
Total score	1.7 ± 1.8	1.0 ± 1.6
≥ 3	136 (27.6%)	67 (16.7%)
*Composite variable 1*^*a*^		
− anxiety − depression	292 (59.4%)	310 (77.5%)
+ anxiety − depression	64 (13.0%)	23 (5.8%)
− anxiety + depression	52 (10.6%)	41 (10.3%)
+ anxiety + depression	84 (17.1%)	26 (6.5%)
*Composite variable 2*^*b*^		
− anxiety − moderate-severe depression	346 (70.3%)	329 (82.3%)
+ anxiety − moderate-severe depression	10 (2.0%)	4 (1.0%)
− anxiety + moderate-severe depression	83 (16.9%)	57 (14.3%)
+ anxiety + moderate-severe depression	53 (10.8%)	10 (2.5%)

^a^− anxiety = GAD < 3, + anxiety = GAD ≥ 3, − depression = PHQ-9 < 10, + depression = PHQ-9 ≥ 10

^b^− anxiety = GAD < 3, + anxiety = GAD ≥ 3, − moderate-severe depression = PHQ-9 < 15, + moderate-severe depression = PHQ-9 ≥ 15

Individuals with anxiety and/or depressive symptoms, based on GAD-2 and PHQ-9 scores, had a considerably lower EQ-5D-3L index and EQ VAS scores compared to those without any symptoms (0.75 and 66.9, respectively): anxiety (0.63, 59.2, respectively), any depressive symptoms (0.64, 63.7, respectively), moderate-severe depressive symptoms (0.64, 62.8, respectively), comorbid anxiety and any depressive symptoms (0.59, 54.6, respectively), and comorbid anxiety and moderate-severe depressive symptoms (0.57, 50.7, respectively) (Table 3).

**Table 3:** EQ-5D-3L in the overall sample and by anxiety and depression sub-groups

	Hospital setting						Community setting					
EQ-5D-3L Components Mean ± SD or N (%)	N	Anxiety/depression dimension			Index Score	VAS	N	Anxiety/depression dimension			Index Score	VAS
		Level 1	Level 2	Level 3				Level 1	Level 2	Level 3		
Overall	492	284 (57.5)	180 (36.4)	29 (5.9)	0.69 ± 0.21	63.4 ± 18.9	400	294 (73.1)	92 (22.9)	16 (4.0)	0.77 ± 0.22	71.4 ± 19.4
Anxiety and Any Depression Sub-Groups												
No anxiety nor any depressive symptoms^a^	292	221 (75.7)	66 (22.6)	5 (1.7)	0.75 ± 0.20	66.9 ± 17.1	310	264 (85.2)	44 (14.2)	2 (0.7)	0.79 ± 0.20	75.5 ± 16.9
Anxiety present, no depressive symptoms^b^	64	28 (43.8)	34 (53.1)	2 (3.1)	0.63 ± 0.21	59.2 ± 18.5	23	11 (47.8)	8 (34.8)	4 (17.4)	0.62 ± 0.24	51.7 ± 23.7
Any depressive symptoms, no anxiety^c^	52	16 (31.4)	28 (54.9)	7 (13.7)	0.64 ± 0.22	63.7 ± 21.3	41	15 (36.6)	22 (53.7)	4 (9.8)	0.63 ± 0.21	64.2 ± 17.6
Anxiety and any depressive symptoms^d^	84	18 (21.7)	50 (60.2)	15(18.1)	0.59 ± 0.20	54.6 ± 20.5	26	2 (7.7)	18 (69.2)	6 (23.1)	0.44 ± 0.26	52.8 ± 21.9
P value		< 0.001			0.137	< 0.001		< 0.001			<0.001	<0.001
Anxiety and Moderate-Severe Depression Sub-Groups												
No anxiety or moderate-severe depressive symptoms^e^	346	244 (70.5)	96 (27.8)	6 (1.7)	0.73 ± 0.20	65.6 ± 17.5	329	273 (83.0)	51 (15.5)	5 (1.5)	0.78 ± 0.21	74.2 ± 18.0
Anxiety present, no moderate-severe depressive symptoms^f^	10	5 (50.0)	4 (40.0)	1 (10.0)	0.57 ± 0.23	63.2 ± 21.1	4	2 (50.0)	1 (25.0)	1 (25.0)	0.59 ± 0.34	44.3 ± 29.6
Moderate-severe depressive symptoms, no anxiety^g^	83	24 (29.3)	45 (54.9)	13 (15.9)	0.64 ± 0.21	62.8 ± 21.0	57	17 (29.8)	34 (59.7)	6 (10.5)	0.59 ± 0.23	61.7 ± 17.5
Anxiety and moderate-severe depressive symptoms^h^	53	10 (19.2)	33 (63.5)	9 (17.3)	0.57 ± 0.21	50.7 ± 19.6	10	0 (0.0)	6 (60.0)	4 (40.0)	0.35 ± 0.26	49.0 ± 29.6
P value		< 0.001			0.003	< 0.001		< 0.001			<0.001	<0.001

^a^GAD-2 < 3 and PHQ-9 < 10; ^b^GAD-2 ≥ 3 and PHQ-9 < 10; ^c^GAD-2 < 3 and PHQ-9 ≥ 10; ^d^GAD-2 ≥ 3 and PHQ-9 ≥ 10

^e^GAD-2 < 3 and PHQ-9 < 15; ^f^GAD-2 < 3 and PHQ-9 ≥ 15; ^g^GAD-2 ≥ 3 and PHQ-9 < 15; ^h^GAD-2 ≥ 3 and PHQ-9 ≥ 15

### Performance of EQ-5D-3L in screening for anxiety symptoms

The EQ-5D-3L anxiety/depression dimension was moderately accurate in screening for anxiety symptoms compared to the GAD-2 (Table 4), whereby the highest performance was in the community setting (AUROC 0.79, 95% CI: 0.74, 0.85) with an optimal performance cut-off point at ≥ 2 (sensitivity 74.6%), and the poorest performance in the hospital setting (AUROC 0.75, 95% CI: 0.70, 0.79). The performance of the EQ-5D-3L index and EQ VAS scores in screening for anxiety were poor with respective AUROC of 0.37 (95% CI: 0.32, 0.42) and 0.40 (95% CI: 0.33, 0.47) in hospital, and 0.27 (95% CI 0.22, 0.33) and 0.33 (95% CI: 0.27, 0.39) in community setting.

**Table 4:** Performance of the EQ-5D-3L in screening for anxiety symptoms (GAD-2 ≥ 3)

Cut-point	Hospital setting (N = 492)		Community setting (N = 400)	
	≥ 2	≥ 3	≥ 2	≥ 3
*EQ-5D-3L Anxiety/depression dimension*				
Sensitivity	**75.0%**	17.7%	**74.6%**	14.9%
Specificity	**69.8%**	98.0%	**82.6%**	98.2%
LR+	**2.48**	9.00	**4.28**	8.28
LR−	**0.36**	0.84	**0.31**	0.87
PPV	**48.0%**	76.6%	**45.0%**	61.2%
NPV	**88.3%**	76.3%	**94.5%**	85.8%
AUROC (95% CI)	0.74 (0.70, 0.79)		0.79 (0.74, 0.85)	
*EQ-5D-3L index score tertiles*				
Sensitivity	52.2%	14.7%	25.4%	4.5%
Specificity	30.3%	62.8%	34.4%	68.3%
LR+	0.75	0.39	0.39	0.14
LR−	1.58	1.36	2.17	1.40
PPV	21.7%	12.8%	6.9%	2.6%
NPV	63.2%	66.6%	70.8%	79.0%
AUROC (95% CI)	0.37 (0.32, 0.42)		0.27 (0.22, 0.33)	
*EQ-VAS score tertiles*				
Sensitivity	52.9%	13.2%	41.8%	10.5%
Specificity	32.4%	73.0%	30.5%	68.0%
LR+	0.78	0.49	0.60	0.33
LR−	1.45	1.19	1.91	1.32
PPV	22.5%	15.3%	10.3%	5.9%
NPV	65.0%	69.5%	73.3%	80.0%
AUROC (95% CI)	0.40 (0.33, 0.47)		0.33 (0.27, 0.39)	

LR+, positive likelihood ratio; LR−, negative likelihood ratio; AUROC, area under receiver operating curve. EQ-5D-3L index score tertiles: 2 = quintile 2, 3 = quintile 3. EQ-5D-3L anxiety/depression dimension: 2 = moderately anxious or depressed, 3 = extremely anxious or depressed. PPV, positive predictive value; NPV, negative predictive value. Bolded values indicate the cut-point that maximizes sensitivity and specificity for each component

### Performance of EQ-5D-3L in screening for depressive symptoms

The EQ-5D-3L anxiety/depression dimension performance in screening for depressive symptoms compared to the PHQ-9 was moderate (Table 5). For any depressive symptoms (PHQ-9 ≥ 10), the highest AUROC was in the community setting (0.78, 95% CI: 0.71, 0.85) with an optimal cut-off point ≥ 2 (sensitivity 73.5%). For moderate-severe depression (PHQ-9 ≥ 15), the highest AUROC was also in the community setting (0.84, 95% CI: 0.73, 0.94) with an optimal cut-off point ≥ 2 (sensitivity 85.7%). The EQ-5D-3L anxiety/depression dimension performed better in screening for moderate-severe depressive symptoms than for mild symptoms. The performance of this dimension in screening for depressive symptoms in the hospital setting was poor. Additionally, the performance of the EQ-5D-3L index and EQ VAS scores in screening for depressive symptoms were also poor in both hospital and community settings.

**Table 5:** Performance of the EQ-5D-3L in screening for depressive symptoms

Cut-point	Hospital setting (N = 492)		Community setting (N = 400)	
	≥ 2	≥ 3	≥ 2	≥ 3
*Any depressive symptoms (PHQ-9* ≥ *10)*				
EQ-5D-3L Anxiety/depression dimension				
Sensitivity	**68.9%**	12.2%	**73.5%**	20.4%
Specificity	**68.8%**	96.2%	**79.6%**	98.3%
LR+	**2.21**	3.24	**3.59**	11.97
LR−	**0.45**	0.91	**0.33**	0.81
PPV	**48.5%**	57.8%	**33.4%**	62.5%
NPV	**83.8%**	72.0%	**95.6%**	89.9%
AUROC (95% CI)	0.70 (0.65, 0.74)		0.78 (0.71, 0.85)	
EQ-5D-3L index score tertiles				
Sensitivity	48.7%	13.5%	28.6%	4.1%
Specificity	28.0%	61.6%	36.7%	69.6%
LR+	0.68	0.35	0.45	0.13
LR−	1.83	1.40	1.95	1.38
PPV	22.4%	13.0%	5.9%	1.8%
NPV	56.1%	62.5%	78.7%	83.9%
AUROC (95% CI)	0.34 (0.29, 0.39)		0.30 (0.24, 0.36)	
EQ-VAS score tertiles				
Sensitivity	51.0%	14.3%	28.5%	6.1%
Specificity	29.0%	71.0%	29.8%	68.5%
LR+	0.72	0.49	0.41	0.19
LR−	1.69	1.21	2.40	1.37
PPV	23.5%	17.4%	5.3%	2.6%
NPV	58.1%	66.0%	75.0%	84.0%
AUROC (95% CI)	0.38 (0.33, 0.43)		0.27 (0.20, 0.33)	
*Moderate-severe symptoms (PHQ-9* ≥ *15)*				
EQ-5D-3L Anxiety/depression dimension				
Sensitivity	**76.2%**	17.5%	**85.7%**	35.7%
Specificity	**62.4%**	95.4%	**75.2%**	97.2%
LR+	**2.03**	3.76	**3.46**	12.56
LR−	**0.38**	0.87	**0.19**	0.66
PPV	**22.6%**	35.4%	**11.4%**	31.6%
NPV	**94.8%**	88.9%	**99.3%**	97.7%
AUROC (95% CI)	0.71 (0.65, 0.77)		0.84 (0.73, 0.94)	
EQ-5D-3L index score tertiles				
Sensitivity	44.4%	11.1%	21.4%	0.0%
Specificity	32.0%	66.1%	39.5%	71.8%
LR+	0.65	0.33	0.35	0.00
LR−	1.74	1.34	1.99	1.39
PPV	8.6%	4.5%	1.3%	0.0%
NPV	80.0%	83.8%	93.3%	95.2%
AUROC (95% CI)	0.34 (0.28, 0.41)		0.27 (0.19, 0.36)	
EQ-VAS score tertiles				
Sensitivity	45.2%	8.1%	28.6%	7.1%
Specificity	32.1%	73.0%	33.6%	70.8%
LR+	0.67	0.30	0.43	0.24
LR−	1.71	1.26	2.13	1.31
PPV	8.8%	4.2%	1.5%	0.9%
NPV	80.3%	84.6%	92.8%	95.5%
AUROC (95% CI)	0.35 (0.29, 0.42)		0.29 (0.17, 0.41)	

LR+, positive likelihood ratio; LR−, negative likelihood ratio; AUROC, area under receiver operating curve; EQ-5D-3L index score tertiles: 2 = quintile 2, 3 = quintile 3. EQ-5D-3L anxiety/depression dimension: 2 = moderately anxious or depressed, 3 = extremely anxious or depressed. PPV, positive predictive value; NPV, negative predictive value. Bolded values indicate the cut-point that maximizes sensitivity and specificity for each component

### Performance of EQ-5D-3L in screening for comorbid anxiety and depressive symptoms

The EQ-5D-3L anxiety/depression dimension performed better in screening for comorbid anxiety and depressive symptoms compared to its performance in screening for each of these symptoms alone. For comorbid anxiety and any depressive symptoms, the anxiety/depression dimension AUROC was 0.86 (95% CI: 0.80, 0.92) in the community setting with an optimal cut-off point ≥ 2 (sensitivity 92.3%) compared to 0.74 (95% CI 0.68, 0.79) in hospital setting (Table 6). For comorbid anxiety and moderate-severe depression, the anxiety/depression dimension AUROC was 0.91 (95% CI: 0.87, 0.95) in the community setting with an optimal cut-off point ≥ 2 (sensitivity 100.0%), compared to 0.73 (95% CI 0.67, 0.79) in hospital setting. The performance of the EQ-5D-3L index and EQ VAS scores in screening for comorbid anxiety and depressive symptoms were poor in both hospital and community settings and for both levels of depressive symptoms.

**Table 6:** Performance of the EQ-5D-3L in screening for comorbid anxiety (GAD-2 ≥ 3) with any depressive symptoms (PHQ-9 ≥ 10), and comorbid anxiety (GAD-2 ≥ 3) with moderate-severe depressive symptoms (PHQ-9 ≥ 15)

Cut-point	HOSPITAL SETTING (N = 492)		COMMUNITY SETTING (N = 400)	
	≥ 2	≥ 3	≥ 2	≥ 3
*Anxiety (GAD-2* ≥ *3) & any depressive symptoms (PHQ-9* ≥ *10)*				
EQ-5D-3L Anxiety/depression dimension				
Sensitivity	**78.3%**	18.1%	**92.3%**	23.1%
Specificity	**65.1%**	96.6%	**77.5%**	97.3%
LR+	**2.24**	5.25	**4.11**	8.63
LR−	**0.33**	0.85	**0.10**	0.79
PPV	**31.3%**	52.0%	**22.2%**	37.3%
NPV	**93.7%**	85.3%	**99.3%**	94.8%
AUROC (95% CI)	0.74 (0.68, 0.79)		0.86 (0.80, 0.92)	
EQ-5D-3L index score tertiles				
Sensitivity	48.8%	11.9%	15.4%	0.0%
Specificity	31.9%	65.0%	38.0%	70.9%
LR+	0.72	0.34	0.25	0.00
LR−	1.61	1.36	2.23	1.41
PPV	12.7%	6.5%	1.7%	0.0%
NPV	75.4%	78.4%	86.7%	91.1%
AUROC (95% CI)	0.36 (0.30, 0.41)		0.24 (0.19, 0.30)	
EQ-VAS score tertiles				
Sensitivity	48.2%	9.6%	26.9%	7.7%
Specificity	31.5%	72.2%	32.4%	70.1%
LR+	0.70	0.35	0.40	0.26
LR−	1.65	1.25	2.26	1.32
PPV	12.5%	6.6%	2.7%	1.8%
NPV	74.9%	79.7%	86.4%	91.6%
AUROC (95% CI)	0.36 (0.31, 0.42)		0.28 (0.19, 0.37)	
*Anxiety (GAD-2* ≥ *3) & moderate-severe depressive symptoms (PHQ-9* ≥ *15)*				
EQ-5D-3L Anxiety/depression dimension				
Sensitivity	**80.8%**	17.3%	**100.0%**	40.0%
Specificity	**62.3%**	95.4%	**74.9%**	96.9%
LR+	**2.14**	3.79	**3.98**	13.00
LR−	**0.31**	0.87	**0.00**	0.62
PPV	**20.3%**	30.8%	**6.8%**	19.1%
NPV	**96.5%**	90.7%	**100.0%**	98.9%
AUROC (95% CI)	0.73 (0.67, 0.79)		0.91 (0.87, 0.95)	
EQ-5D-3L index score tertiles				
Sensitivity	45.3%	11.3%	10.0%	0.0%
Specificity	32.8%	66.5%	39.7%	72.1%
LR+	0.67	0.34	0.17	0.00
LR−	1.67	1.33	2.26	1.39
PPV	7.4%	3.9%	0.3%	0.0%
NPV	83.5%	86.3%	96.0%	97.5%
AUROC (95% CI)	0.35 (0.29, 0.42)		0.23 (0.16, 0.31)	
EQ-VAS score tertiles				
Sensitivity	40.4%	3.9%	28.6%	14.3%
Specificity	32.0%	72.8%	34.2%	71.3%
LR+	0.59	0.14	0.43	0.50
LR−	1.87	1.32	2.09	1.20
PPV	6.6%	1.7%	0.8%	0.9%
NPV	81.9%	86.5%	96.3%	97.8%
AUROC (95% CI)	0.32 (0.26, 0.38)		0.32 (0.12, 0.52)	

LR+, positive likelihood ratio; LR−, negative likelihood ratio; AUROC, area under receiver operating curve. EQ-5D-3L index score tertiles: 2 = quintile 2, 3 = quintile 3. EQ-5D-3L anxiety/depression dimension: 2 = moderately anxious or depressed, 3 = extremely anxious or depressed. PPV, positive predictive value; NPV, negative predictive value. Bolded values indicate the cut-point that maximizes sensitivity and specificity for each component

## Discussion

In this study, we found that the EQ-5D-3L anxiety/depression dimension performed well in screening for anxiety and depressive symptoms in adults in the community setting; it was less useful in the hospital setting, particularly at the time of discharge. Additionally, the screening performance of this dimension was better when anxiety and depressive symptoms were both present than either symptom alone. Further, this dimension was more accurate for “moderate-severe” levels of depressive symptoms, either alone or with anxiety, than for “any” levels of depressive symptoms. The screening performance of the EQ-5D-5L anxiety/depression dimension was similar to previously validated, but separate, screening measures for each of the set of symptoms. Finally, we found that the EQ-5D-3L index and EQ VAS scores generally had poor performance in screening for anxiety and/or any level of depressive symptoms in both hospital and community settings.

The proportion of patients reporting problems on the anxiety/depression dimension (levels 2–3) in hospital and community settings were 42% and 27%, respectively; higher than the Alberta population norm, 23% [44]. Lower EQ-5D-3L index and EQ VAS scores were observed in patients with anxiety and depressive symptoms and more considerably among the co-morbid anxiety and depressive symptoms groups, with the lowest index and EQ VAS scores observed at 0.45 and 49.0, respectively. These overall scores were nonetheless also picking up the considerable physical health problems in this sample. It is evident that there is an emotional and overall health-related quality of life burden associated with anxiety and/or depressive symptoms, especially when compared to the overall Alberta adult population norms of the EQ-5D-3L index and EQ VAS scores of 0.93 and 78.3, respectively [44].

Several studies have supported the use of the EQ-5D-3L for measuring health status in individuals with mental health problems [14–16], but only one other study has shown that the EQ-5D-3L is a useful screening tool for anxiety and depressive symptoms in the general population [17]. Supina et al. (2007) examined the ability of the EQ-5D-3L in differentiating those with a clinical diagnosis of major depressive disorder and/or an anxiety disorder. The authors found that major depression disorder is slightly more predictive of problems reported (levels 2–3) on the anxiety/depression dimension than anxiety alone, and the co-morbidity of depression and anxiety resulted in a significantly greater likelihood of respondents reporting problems. Additionally, Al Sayah et al. (2018) investigated the performance of the EQ-5D-5L in screening for anxiety and depressive symptoms in type 2 diabetes patients in an outpatient community setting. They found that the EQ-5D-5L anxiety/depression dimension performed very well in screening for anxiety (AUROC: 0.89), depressive symptoms (any: 0.88, moderate-severe: 0.90), and comorbid anxiety and depressive symptoms (any: 0.92, moderate-severe: 0.92) [10]. These two studies provide similar evidence to our study and support our findings that the EQ-5D-3L is more appropriate in a community setting. Preference based measures, like the EQ-5D-3L, are commonly used for economic evaluations, but are increasingly being used in community health care settings for quality improvement purposes, patient management, and surveillance of population health [10]. Routine mental health screening using a generic PROM, like the EQ-5D-3L, could also be implemented in addition to these large-scale community health applications [10].

Our findings suggest that the EQ-5D-3L, specifically the anxiety/depression dimension, would be more suitable in screening for anxiety and/or depressive symptoms in an outpatient community setting rather than the hospital setting. During and after emergencies, people are more likely to suffer from a range of mental health problems, often related to poor adjustment to hospitalization distress [45]. Some people develop new mental disorders after an emergency, while others experience temporary mental distress [11]. Our findings suggest that patients experienced more anxiety and depressive symptoms in hospital (at discharge) than in the community setting (90-days post-discharge), possibly due to the distress of hospitalization. For depression and anxiety diagnoses, anxiety and depressive symptoms need to persist for an extended period of time, usually at least 2 weeks, which is the reference period on the GAD and PHQ measures. The EQ-5D-3L reference period “today” may have impacted the prevalence of positive anxiety and/or depression screens at the time of discharge, as hospitalization is a disruptive, worrisome event that could influence patients’ reporting of anxiety/depression. Further, we likely see the best screening accuracy of the EQ-5D-3L anxiety/depression dimension in the community setting because those who experienced anxiety and/or depressive like symptoms at discharge only in relation to hospitalization, no longer have these symptoms in the community, 90-days post-discharge, and therefore do not report any problems on the EQ-5D-3L anxiety/depression dimension. Whereas, those who have anxiety and/or depressive symptoms unrelated to the hospitalization were likely to continue to report problems and screen positive in the community setting.

Within both the hospital and community settings, the screening performance of the anxiety/depression dimension was better when anxiety and depressive symptoms were both present than either symptom alone. Previous studies have examined how composite EQ-5D dimensions, a combination of two separate but related items, such as the anxiety/depression dimension, are interpreted by respondents [46, 47]. Tsuchiya et al. (2019) found that what respondents have in mind when valuing “extreme anxiety or depression” is that both conditions are significantly worse than extreme anxiety on its own [46]. Furthermore, depression was perceived to be worse than anxiety at the same level. Our study findings are in accordance with these results given that the sensitivity is stronger for anxiety than depression and strongest when both anxiety and depression are present. However, McDonald et al. (2020) found that respondents interpret the anxiety/depression dimension as “the component of anxiety and depression with the most severe reported problems” [47]. Nonetheless, respondents seem to consider both anxiety and depression when responding to the anxiety/depression dimension which is not the case of the pain/discomfort dimension where respondents mainly use this dimension to only report pain [47].

Although mental disorders, depression in particular, have been identified as the most common diagnosis among people hospitalized with physical diseases [48], screening is not regularly done among hospitalized patients. Guidelines differ between agencies despite the availability of suitable screening tests [10] and are specified for the primary care outpatient setting. In the hospital setting, there is a pressure to provide cost and time-efficient care that discharges patients as quickly as possible [9]. It is suggested that if mental health screening were to become routine in hospital settings, screening tools need to be sensitive, specific, brief, and suitable for self-administration by patients or health care providers [9]. While, the EQ-5D-3L meets most of those criteria, the reference period “today” remains an issue for the hospitalized patient population. Mental health screening using the EQ-5D-3L in the hospital setting may be less useful in identifying long-term mental health issues and further screening in the community after discharge would be necessary.

This study has a few limitations that should be considered in interpreting its results. First, anxiety and depressive symptoms were measured by self-report. Although GAD-2 and PHQ-9 are widely used and established screening measures of anxiety and depressive symptoms, respectively, comparing the performance of the EQ-5D-3L to clinical assessments of these symptoms would enhance the robustness of these analyses. Second, the EQ-5D-3L was not developed for clinical screening or diagnosis. The reference period of the tool is “today”, and each dimension asks about the severity of problems, not their frequency or impact on life. These characteristics may affect the sensitivity and specificity of the tool. For instance, disorders such as generalized anxiety tend to be highly persistent, whereas others, such as panic disorder, tend to be characterized by episodic symptoms [17]. Third, the Canadian EQ-5D-3L uses the wording ‘I am not/moderately/extremely anxious or depressed’. There is possible bias that respondents interpret this as a clinical diagnosis rather than a feeling, therefore, underreporting anxiety/depressive symptoms. Fourth, some of the examined groups within the overall sample had small sample sizes, also limiting the analysis. And lastly, this study included predominantly Caucasian older-aged adults in Alberta, Canada, limiting the generalizability of the results to similar populations.

## Conclusions

Our findings suggest that the EQ-5D-3L anxiety/depression dimension could be a useful screening tool for anxiety and depressive symptoms, compared to other self-report screening tools, in community settings. The performance of this tool in the hospital setting was poor. While screening does not replace clinical diagnosis, this tool may help in identifying patients with anxiety and/or depressive symptoms that otherwise may not be identified. Examining the screening performance of EQ-5D-3L and other patient-reported outcome measures in other populations and settings is warranted.

## Data Availability

The data that supports the findings of this study are available from the University of Alberta but restrictions apply to the availability of these data, which were used under license for the current study, and so are not publicly available. Data are however available from the authors upon reasonable request and with permission of the University of Alberta.

## Abbreviations

ANOVA: Analysis of variance
AUROC: Area under receiver operating curve
EQ VAS: EuroQol Visual Analogue Scale
GAD-2: Generalized Anxiety Disorder 2-item
PHQ-9: Patient Health Questionnaire 9-item
PROMs: Patient-reported outcome measures
QALY: Quality-adjusted life year
